# Cardiovascular Data Abstraction Centralization: Impact on Patient Care, Operational Efficiency, and Strategic Decision-Making

**DOI:** 10.1016/j.jaccas.2026.109413

**Published:** 2026-09-16

**Authors:** Sheila B. Nichols, Gwynlynn Weil, Barbara Sanders

**Affiliations:** Quality, Analytics and Reporting Department, Allegheny Health Network, Pittsburgh, Pennsylvania, USA

**Keywords:** cardiovascular registries, centralization, data abstraction, health care management, operational efficiency, quality improvement

## Abstract

**Background:**

Fragmented cardiovascular (CV) data abstraction across Allegheny Health Network led to inconsistent data quality, resource inefficiencies, and limited real-time insights, impeding patient care and strategic decision-making.

**Project Rationale:**

To address these challenges, Allegheny Health Network initiated a project to centralize CV registry abstraction, aiming to standardize processes, improve data integrity, enhance operational efficiency, and provide timely, actionable insights to clinicians.

**Project Summary:**

A current state assessment identified 8 CV registries and 11.5 full-time equivalents primarily engaged in abstraction across 5 facilities. A new corporate department was established, a dedicated manager and coordinator hired, nonabstraction tasks reallocated, and standardized workflows implemented. This led to significant reductions in abstraction time, improved accuracy, and enhanced reporting.

**Take-Home Messages:**

Centralized data abstraction drives significant efficiency gains, improves data quality, and empowers clinical and strategic decision-making in complex healthcare networks.

The modern health care landscape increasingly relies on robust, accurate, and accessible data to drive improvements in patient care, optimize operational efficiency, and inform strategic planning. Cardiovascular (CV) registries, such as those within the National Cardiovascular Data Registry program, are fundamental to elevating cardiac care, as they aggregate real-world data to benchmark performance, track patient outcomes, and ensure adherence to evidence-based guidelines that power continuous quality improvement initiatives across health systems.^1^ However, in large, integrated health networks, the management of these registries can often become decentralized, leading to inconsistencies in data collection, inefficient resource allocation, and a lack of actionable insights. This fragmentation can hinder a network's ability to fully leverage its data for continuous quality improvement and evidence-based decision-making. This article describes a comprehensive project undertaken by Allegheny Health Network (AHN) to centralize its cardiovascular registry data abstraction, detailing the rationale, methodology, outcomes, and implications of this strategic initiative.

**Take-Home Messages**

• Centralized, standardized data abstraction is a powerful lever for improving patient care, driving operational efficiency, and empowering strategic decisions across a health care system.
• Investing in dedicated resources and standardized processes for data management yields significant returns in data quality, cost savings, and actionable insights.

## Project Rationale

Before this project, AHN's cardiovascular data abstraction was highly decentralized, characterized by diverse approaches across 5 facilities. A detailed current state assessment conducted in early 2019 revealed several critical challenges that prompted the need for a centralized model. These included a lack of consistent contracting for registries, significant variability in data collection methodologies, and the absence of a standardized quality control process. Furthermore, resources for data analysis were insufficient, leading to underutilized data and limited capacity for researching best practices and recommending evidence-based actions.

The dispersed model also exposed vulnerabilities in staffing, with a lack of cross-coverage for vacations, leaves, and retirements across 48 individuals involved in abstraction roles spanning various departments, job titles, and credentials (eg, registered nurses, nurse practitioners, physician assistants, perfusionists). There was limited alignment with the central CV Institute, inconsistent organizational structures, and a notable absence of real-time data for decision-making. The project's intent was to address these inefficiencies by reorganizing CV registry abstraction into a corporate department, matrixed back to the CV Institute. The overarching goals were to maintain operations, provide analytics, deliver real-time feedback to clinicians, and ultimately enhance patient outcomes and operational efficacy through standardization and expertise building.

## Project Description

The centralization of CV registries was initiated in early 2019 with a comprehensive current state assessment led by the director of clinical data abstraction. This involved interviews and surveys to identify all relevant registries and personnel involved in abstraction, analytics, and reporting functions. Eight CV registries across 5 hospitals were targeted for centralization. Of the 48 individuals identified as having some involvement with CV registries, 11.5 full-time equivalents (FTEs) had a primary role in data abstraction, submission, and reporting.

A dedicated manager for the new centralized team was hired in August 2020. This manager conducted further interviews and surveys to ascertain the specific roles, titles, and skill sets of all personnel. A detailed needs assessment was performed, considering registry abstraction times and case volumes for each procedure type. This assessment determined a need for 12.4 FTEs, revealing a 0.9-FTE deficit compared with the identified 11.5 FTEs available for transition (Figure 1). Consequently, the transition of the vascular registry was strategically deferred until efficiencies were gained within the initial centralized team.

**Figure 1:**
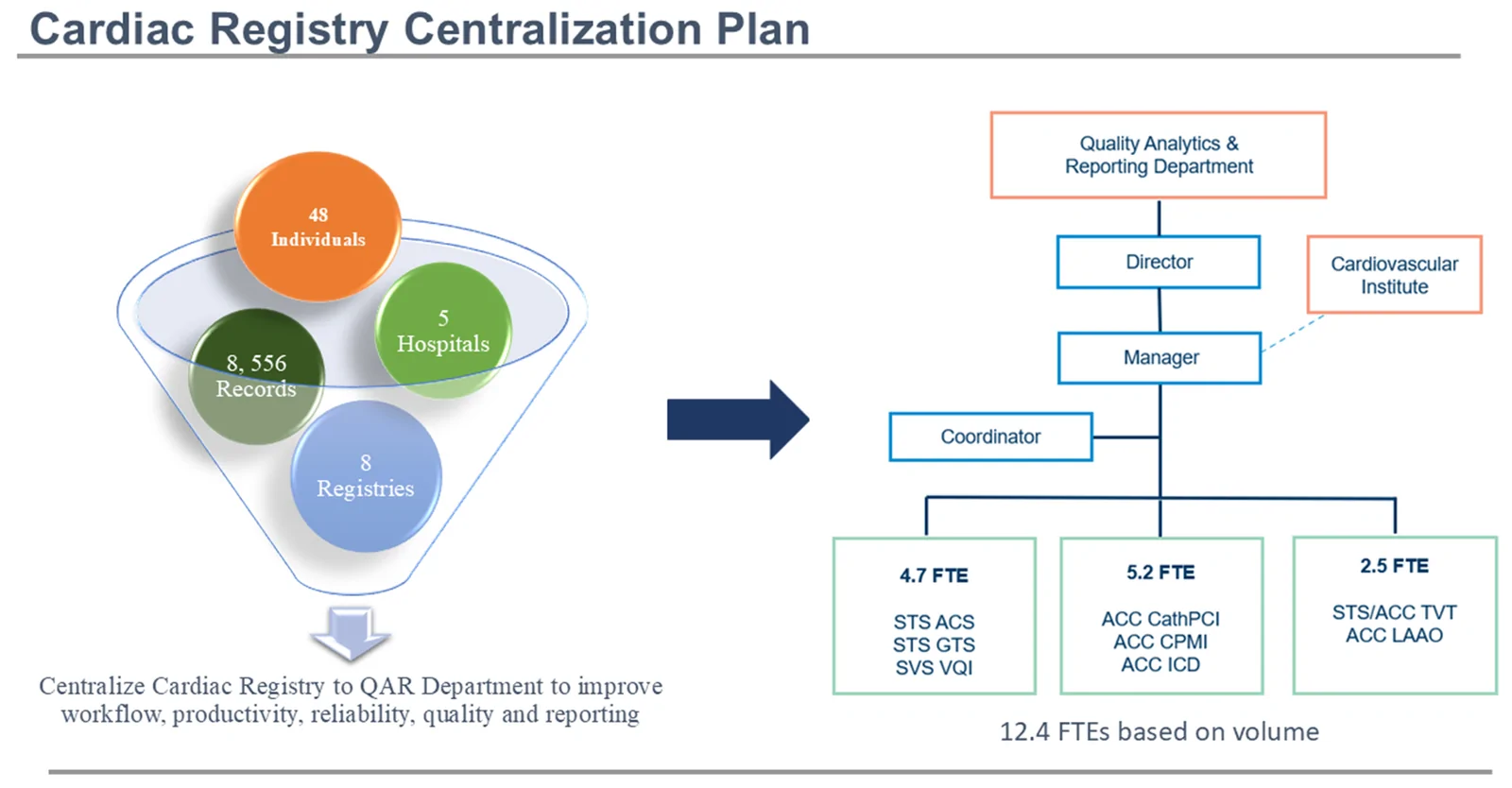
Centralizing the Abstraction Team Required 12.4 FTE Employees ACC = American College of Cardiology; ACS = adult cardiac surgery registry; CathPCI = CathPCI registry; CPMI = Chest Pain-MI registry; CV = cardiovascular; FTE = full-time equivalent; GTS = general thoracic surgery registry; ICD = ICD registry; LAAO = LAAO registry; STS = society of thoracic surgery; SVS = society for vascular surgery; TVT = TVT registry; VQI = vascular quality intiative.

Extensive review of existing workflows led to the identification and re-allocation or discontinuation of tasks that fell outside the core data abstraction scope, transferring them to appropriate hospital staff. To bolster ongoing quality and education, a coordinator position was added to facilitate continuous learning opportunities and consistent inter-rater reliability (IRR) evaluations. The newly formed team officially transitioned under the centralized manager in June 2021. To ensure continuity of work and support educational needs, the acquisition of data abstraction responsibilities was staggered. Initially, 5 registries moved to the centralized team, followed 2 months later by the addition of 2 more registries and the formal handover of all remaining nonregistry work.

## Project Deliverables

The primary deliverables of the centralization project focused on establishing a robust and efficient operational model for CV registry abstraction:

Centralized Team and Structure: Creation of a dedicated corporate department for CV registry abstraction, with a clear management structure and matrix reporting to the CV Institute.

Standardized Workflows and Protocols: Implementation of uniform abstraction guidelines, standardized abstraction documents, and clear protocols across all centralized registries.

Enhanced Quality Control: Establishment of consistent IRR evaluations and a dedicated role to oversee ongoing education and quality assurance.

Improved Staffing Model: Development of a staffing model that ensures cross-coverage for absences and leverages remote work capabilities to expand the talent pool.

Timely Reporting and Feedback: Implementation of strict timeline standards for abstraction (1-2 weeks postdischarge) and data submission (weekly), alongside weekly outlier investigations and regular communication channels with clinicians and leadership.

Actionable Insights: Dissemination of biweekly outlier reports, monthly morbidity and mortality reports for surgical registries, and quarterly published reports to various network quality meetings, facilitating quality improvement projects and sharing of best practices.

## Project Outcome, Impact, and Future Directions/Next Steps

The centralization of AHN's CV registry abstraction yielded significant positive outcomes, impacting efficiency, data quality, and strategic capabilities.

### Efficiency gains

A follow-up time study 6 months after implementation revealed a 16% improvement in abstraction time per case, with an average reduction of 9.72 minutes from a baseline of 62.58 minutes (Figure 2). Considering an estimated 2021 case volume of 6,351 for the studied registries, this translates to an annual savings of 1,029 hours. At a national average abstractor cost of $42 per hour,^2^ this represents potential direct labor cost saving of more than $43,218. These figures highlight the substantial operational efficiencies achieved through standardization and focused management. The result of this success allowed for the strategic integration of the vascular registry, which was initially deferred, to further leverage the established efficiencies. Furthermore, the efficiencies demonstrated have allowed expansion of services by adding an additional hospital volume and 3 additional CV registries, showcasing our commitment to sustainable growth and optimized resource utilization.

**Figure 2:**
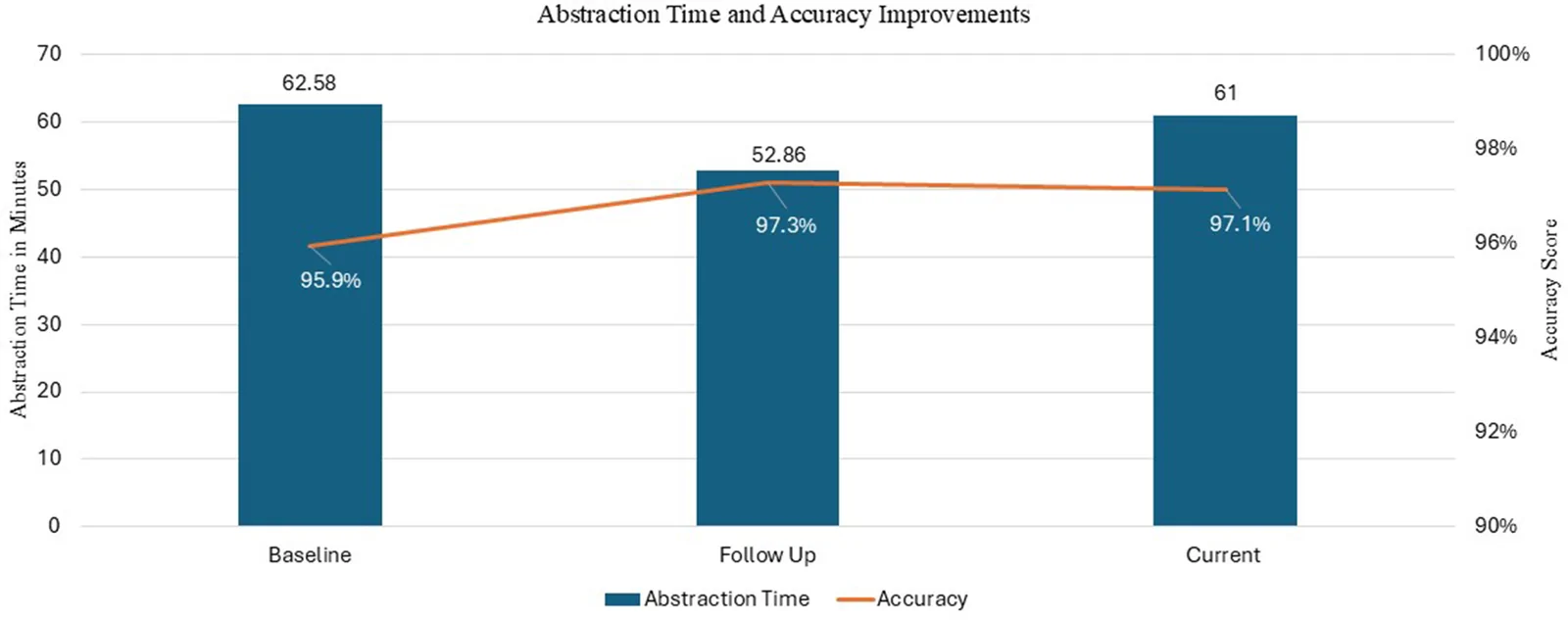
Abstraction Time and Accuracy Improvements Were Measured as an Aggregate Score of 6 Cardiovascular Registries

### Improved accuracy and reporting

The centralized team achieved an aggregate data accuracy score of 97.28%, signifying a marked improvement in data integrity from the baseline score of 95.94% (Figure 2). This enhanced accuracy, combined with the implementation of real-time data for clinical decision-making, weekly outlier investigations, and regular comprehensive reports, provided clinicians and leadership with unprecedented insights. These mechanisms allowed for proactive validation of outcomes and informed quality improvement initiatives.

### Employee satisfaction

Despite the significant organizational change, the project resulted in notable improvements in employee engagement and satisfaction scores within the new centralized team. The remote work model broadened the hiring pool, allowing for the recruitment of diverse talent and better resource allocation.

### Future directions

Continuous process refinement, exploration of automation opportunities within the abstraction workflow, and expansion of the centralized model to other clinical registries within the network represent key future directions. The aim is to continue fostering a culture of data-driven quality improvement and extend the benefits of centralized data management across AHN (Figure 3).

**Figure 3:**
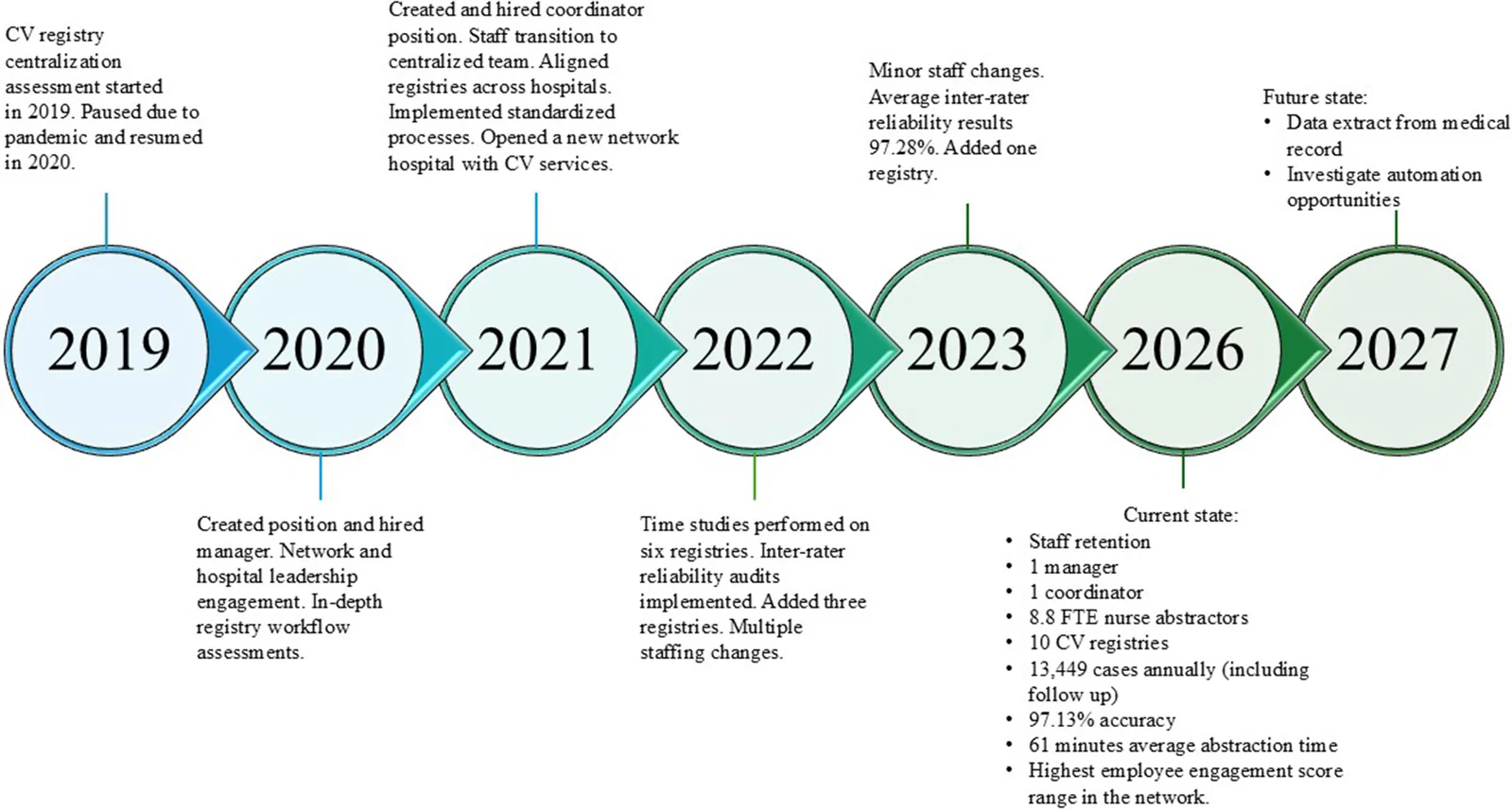
Project Timeline From Inception to Future State Abbreviations as in Figure 1.

## Discussion

The successful centralization of cardiovascular data abstraction at AHN provides a compelling case study for large health care systems grappling with fragmented data management. The initial assessment clearly articulated the need for change, revealing inefficiencies born from inconsistent processes, resource constraints, and a lack of unified oversight. By creating a dedicated corporate department and implementing standardized workflows, AHN effectively addressed these foundational issues.

A critical aspect of this success was the strategic approach to transition, including the staggered acquisition of registries and the re-allocation of nonabstraction tasks. This meticulous planning ensured continuity of essential data collection during the transformation period. The addition of a role for education and IRR was instrumental in maintaining high data quality and leading directly to the impressive 97.28% accuracy rate. Furthermore, the ability to leverage remote work not only broadened the talent pool but also contributed to improved employee satisfaction, likely due to increased flexibility and focused work environments.

The quantifiable benefits, particularly the 16% reduction in abstraction time and more than $40,000 in estimated annual savings, underscore the tangible economic impact of such initiatives. Beyond financial benefit, the enhanced reporting mechanisms—including weekly outlier investigations and regular quality reports—have empowered clinicians with more timely and reliable data, facilitating proactive quality improvement and informed clinical decision-making. This project demonstrates that investing in data infrastructure and centralized management can yield substantial returns in both operational efficiency and clinical quality. The model also offers a blueprint for scaling National Cardiovascular Data Registry data abstraction operations, particularly valuable in contexts of postmerger integration in which consistency and efficiency are paramount.

## Conclusions

The centralization of CV registry abstraction at AHN has proven to be a highly successful strategic initiative. It has effectively transformed a fragmented and inefficient system into a cohesive, high-performing department, leading to significant improvements in operational efficiency, data integrity, and the utility of data for clinical decision-making. This project exemplifies the value of a well-planned and executed centralization process in addressing systemic challenges within a complex health care network, highlighting the importance of continuous improvement and strategic alignment.

## Funding Support and Author Disclosures

The authors have reported that they have no relationships relevant to the contents of this paper to disclose.
